# High-efficient synthesis of carbon quantum dots from orange pericarp as fluorescence turn-on probes for Ca^2+^ and Zn^2+^ ion detection and their application in trypsin activity characterization

**DOI:** 10.22038/IJBMS.2022.67323.14758

**Published:** 2023-02

**Authors:** Shakiba Tolou-Shikhzadeh-Yazdi, Niloofar Shakibapour, Sare Hosseini, Parisa Mokaberi, Bizhan Malaekeh-Nikouei, Jamshidkhan Chamani

**Affiliations:** 1 Department of Biology, Faculty of Sciences, Mashhad Branch, Islamic Azad University, Mashhad, Iran; 2 Cancer Research Center, Mashhad University of Medical Sciences, Mashhad, Iran; 3 Nanotechnology Research Center, Pharmaceutical Technology Institute, Mashhad University of Medical Sciences, Mashhad, Iran

**Keywords:** Carbon quantum dots, Enzyme activity, Field emission scanning - electron microscope, Hydrothermal method, Ions detection, Orange pericarp

## Abstract

**Objective(s):**

In this work, we propose an efficient preparation process for the synthesis of natural carbon quantum dots (NCQDs) by the usage of orange pericarp as the carbon natural resource, which is performed through hydrothermal treatment and top-down approaches.

**Materials and Methods:**

The structural, morphological, average size, and optical properties of synthesized NCQDs were investigated via dynamic light scattering (DLS), transform infrared spectroscopy (FTIR), X-ray diffraction (XRD), transmission electron microscopy (TEM), atomic force microscopy (AFM), field emission scanning electron microscope (FESEM), energy dispersive x-ray spectroscopy (EDX), ultraviolet-visible spectroscopy (UV-Vis), and fluorescence PL spectra.

**Results:**

The shape of obtained NCQDs was observed to be spherical in the results of TEM analysis with an average size of 6–7 nm which confirms NCQDs essence. The signs of a strong peak (absorption) at around 282 nm throughout the UV-vis spectrum have been detected. The provided FTIR spectroscopy confirmed the existence of functional groups above the NCQDs surface. Furthermore, the surface charge of -11 mV through the obtained zeta potential regarding the synthesized NCQDs has been identified. MTT assay on mouse colon carcinoma cells (C26) demonstrated the lack of any signs of toxicity in NCQDs.

**Conclusion:**

The obtained NCQDs contain high photo-stability, excellent PL activity, and efficient fluorescent emission based on excitation. The results of kinetic studies revealed the ability of NCQDs to inhibit trypsin activity in a non-competitive model, which qualifies them as potent inhibitors and quenchers of trypsin. It can be suggested that the synthesized NCQDs have the potential of functioning as a sustainable and eco-friendly source for various applications such as sensors for detecting Ca^2+^ and Zn^2+^ and trypsin biosensor for determining enzyme activity.

## Introduction

Nowadays, the unbelievable capacity of nanotechnology can be vividly observed due to the spreading of its impact from basic science to prototype implementations. In general, substances that are shrunk to the level of nanoscale are the main topic of this field since they contain different composition sizes and shapes. It is notable how the nanometer size of these products results in the creation of distinctive features with the ability to be altered in accordance with the aimed objection (1). The occurrence of breakthroughs in nanotechnology is often caused by nano-materials (2), while regardless of their origins, nano-particles are usually associated with unrepeated qualities (3). Standing as a relatively new member of this field, Luminescent carbon dots (C-dots) proved to be applicable in a wide range of health, energy, and environmental applications (4). Being labeled as a novel section of nano-sized carbonic materials Carbon quantum dots (CQDs) contains sizes below 10 nm and consist of quasi-spherical and discrete nano-particles, which are nowadays recognized as remarkable products. CQDs were initially discovered in the course of purifying and isolating single-wall carbon nano-tubes through the employment of gel electrophoresis in 2004 (5,6), which were named afterward in 2006 by Sun *et al.* with the label «carbon quantum dots” (7); this material is also recognized as carbon dots (CDs or C-dots) or carbon nano-dots (CNDs) (8). Being manifested as nanostructures, CQDs are composed of carbon atoms in the core, while the surface can be passivated or functionalized through organics or biomolecules, which are commonly found to contain a size of below 10 nm (9). These small carbon nano-particles are accommodated with distinctive qualities including fine conductivity, high chemical stability, environmental friendliness, broadband optical absorption, low toxicity, strong photoluminescence (PL) emission and optical properties, and effortless cheap synthesis at large-scale, which is corresponding to quantum dots. The diversity of CQDs characteristics is dependent on their components and construction, while the amazing solubility in water and biocompatibility of this material is mostly provided by the carboxyl moieties that exist on its surface. Although the high toxicity of CQDs even at low levels cannot be denied, which is caused by the existing heavy metals, the interest of many is invested in this product due to its impressive morphology, size, and stability. In the company of commonly applied graphene quantum dots, cadmium quantum dots, and carbon dots (CDs), CDs are thoroughly affirmed as nanostructures due to certain advantages including the high rate of being cost-effective, simple synthesis in large scales, satisfying chemical composition, easy functionalization, harmonizing fluorescence emission, high solubility, and photochemical stability (10-12). Furthermore, carbon is categorized among the principal green elements on earth. CQDs, luminescent semiconductor carbon nano-particles, stand as the most attainable allotropes of carbon-based nanomaterials. Due to their smaller sizes, distinctive qualities can be exhibited by CQDs, including high photostability and high quantum yield, broad absorption spectra, strong fluorescent lifetime, simple functionalization with organic molecules, emission tenability, and chemical inertness (13-18). As stated before, the highly crystalline and fluorescent CQDs can bestow the required capacity for being applied in bio-sensors, bio-medical imaging, drug delivery, and solar cell applications. In addition, there is a vast range of applications that can exert these features including humidity sensing, fluorescence detection, fluorescent imaging, bacterial labeling, electronics, capturing the images of bacterial and fungal cells, light emitting diodes, and DNA detection (19-27). According to another investigation, the applicability of C-dots for theranostic purposes can furnish the performance of bio-labeling and thus, provide a speedy recovery through early diagnosis and disease management (28, 29). Among the other advantages of C-dots, one can mention the smaller number of side effects, cheap production, and control of intellectual property rights in regards to performing a triumphant theranostic implication (30, 31). A noticeable amount of interest has been invested in the synthesis of CQDs regarding economical and green production concepts of chemistry in recent years. Discovering inexpensive and reachable organic resources that could function as carbon precursors for composing CQDs remains to be a challenge for researchers (32). There are a number of different top-down and bottom-up approaches available for the synthesis and development of CQDs such as hydrothermal, microwave, ultrasonic, laser ablation, electrochemical oxidation, and chemical oxidation treatments (33). However, we decided to perform the synthesis of CQDs through a hydrothermal route and top-down techniques that involved the usage of orange pericarp as a natural precursor (34). Hydrothermal treatment is an applicable choice for preparing novel carbon products due to certain advantages including low cost, cheap apparatus, fine selectivity, and nontoxic routes (35). On the other hand, top-down techniques are exerted to synthesize CQDs by macroscopic carbon structures, such as graphite, activated carbon, and carbon amorphous, to a nano-crystalline core with predominantly sp2 carbon, the lattice spacing of which are consistent with graphitic or turbostratic carbon (36-38). Therefore, it can be stated that in this approach, CQDs are generated from relatively macroscopic carbon sources. It is remarkable how most of the available natural products can be used as a matrix in this method (39). Many different carbon sources have been reported to be applicable for the synthesis of C-dots including lamp soot, food caramels, soya milk, orange juice, eggshell, protein (bovine serum albumin), watermelon, mango peel, sweet lemon peel, durian shell, and pomegranate waste peels (40-44). Although there are certain review articles published on the topic of CQDs that have emphasized the synthesizing procedures, features, surface engineering, and applications of this material in biomedicine and energy conversion (45-48), this report introduces a novel nano-particle that can be produced via a facile route. For the very first time, we succeeded in synthesizing CQDs through the adoption of a green hydrothermal method, and this resulted in a product with good biocompatibility, better stability, and low toxicity from orange pericarp, which can be exerted in medical therapy, bio-imaging, cell treatment, and detecting the path of drug delivery. Furthermore, the obtained CQDs were assayed in terms of structural and optical properties, as well as cytotoxicity and morphological aspects. The applied mechanism in this article can be considered a novel approach for performing the green synthesis of CQDs and so far, there is no other similar discussion available in this field. The structure and fluorescent properties of so-prepared NCQDs were systematically characterized. The synthesized NCQDs exhibit no cytotoxicity, good water solubility, considerable quantum yields, and good photostability. Moreover, some applications of NCQDs as biosensors in ion-detection (Ca^2+^ and Zn^2+^) and trypsin activity determination have been revealed.

## Materials and Methods

***Materials***

We procured fresh orange pericarp from a local juice shop in Mashhad, Iran. Ethylenediaminetetraacetic acid (EDTA) and Sodium hydrogen carbonate were used to prepare the dialysis tube, which was purchased from Merck Co and DRM-CHEM. Trypsin was prepared in phosphate buffer (pH 7.5 and 50 mM) and the NCQDs were dissolved in double-distilled water (1 mM) as a ligand. In addition, the BApNA stock solution (3 mM) was formulated in DMSO. All of the required solutions were freshly produced; and all of the performed experiments involved the usage of distilled water.

One-way analysis of variance (ANOVA) by US comparison test was performed to compare the effect of treatments using Minitab software 17 version, *P*<0.05 was considered significant. The data were recorded as mean±standard deviation.

***Methods***

*Preparation of orange pericarp*

Orange pericarp was thoroughly cleaned with distilled water to remove the dust particles. Then, we had them chopped and completely dried in an open atmosphere for 1 week. The prepared orange pericarp was required to be ground to powder before beginning the NCQDs production procedure.

*Synthesis of NCQDs from orange pericarp*

**Scheme 1:**
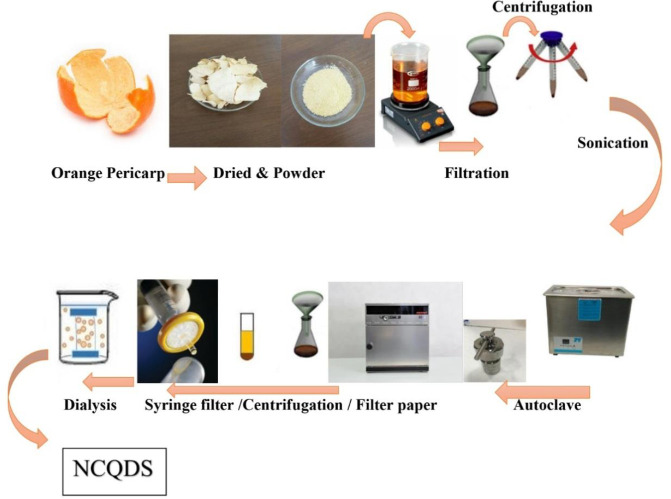
natural carbon quantum dots (NCQDs) synthesis from orange pericarp

NCQDs were synthesized via a facile hydrothermal procedure that involved the usage of orange pericarp as the carbon source. Initially, 2.5 gr of powder and 100 ml of double-distilled water were placed in a beaker and stirred at 60 ^°^C for 2 hr. Then, the solution was poured into a strainer to be centrifuged at 8317.6 g for 10 min at 10 ^°^C for the purpose of dispatching the large particles. The obtained supernatant liquid was collected and sonicated for 10 min. A 100 ml Teflon-lined stainless-steel autoclave was exerted to heat the solution in an oven for 4 hr at 140 ^°^C. Once the reaction was completed, we allowed the autoclave to naturally cool down to ambient temperature and shifted its contents into a strainer again. In order to separate the precipitate, the resultant was centrifuged again at 8317.6 RCF for 10 min at 10 ^°^C. Thereafter, the supernatant liquid was passed through a filter paper and the filtered liquid was filtered again by the usage of a sterile syringe filter unit (0.22 µm) to achieve more purification. The obtained product was sonicated for 30 min in order to be dispersed and reduced in size. Eventually, we dialyzed the filtrate through a dialysis tube for 24 hr (35 kDa, molecular weight cutoff) to provide a purified and homogenous NCQDs solution without any insoluble substances. The final product was vacuum freeze-dried to produce NCQDs powders for further usage and investigation (Scheme 1).

***Characterization studies***

*Fourier transform infrared spectroscopy*

To characterize the surfaces of NCQDs functional chemical groups, FTIR spectroscopy analysis seemed to be a suitable option for identifying the vibrational bond energies of our sample. Fourier Transform Infrared (FT-IR) spectra were recorded at room temperature on a Thermo Nicolet (FTIR, Avatar 370, USA), with a spectral range of 4000-400 cm^-1^ and a resolution of 4 cm^-1^.

*X-ray diffraction*

The degree of crystallinity and phase purity of NCQDs were determined on an X-ray diffraction (XRD) powder spectrometer (X’PertPrp, Panalytical, Netherlands). The synthesized natural carbon quantum dots were equipped with CuKα radiation (wavelength λ=1.54 Å) at the voltage of 40 kV and the current of 30 mA, which were measured in scattering angle 2θ range of 10-90 ^°^. We calculated the average crystal size (D_hkl_) in the system of mono-crystalline through the usage of Debye-Scherrer equation, (this formula was named by Paul Scherrer) Eq. 1: 

D_hkl_= 0.9 λ / β_1/2_ cosθ (1)

The Scherrer parameters were labeled as follows: D_hkl_ represents the mean size of crystalline domains; λ stands for the X-ray wavelength (λ=1.54Å), θ would be the Brigg angle, k represents a dimensionless shape factor (0.9), and β_1/2_ would be the line broadening at half the maximum intensity (FWHM).

*Thermogravimetric analysis*

Thermogravimetric analysis (TGA) and derivative, Thermogravimetric (DTG), are two typical methods exerted to evaluate the thermal stability of NCQDs, which is measured by the utilization of (TGA-50, Shimadzu, Japan) instrument. A thermogravimetric analyzer was applied to determine the induced changes in physical and chemical properties such as weight loss, moisture loss, pyrolysis, decarboxylation, thermal-oxidative, and vaporization. To evaluate the degradation of the sample, it was required to analyze the induced changes in its properties at a heating rate of (10 ^°^C/min), representing the constant mass loss, in the air atmosphere. Observing a slight mass loss that would be parallel to little or no rise in the TGA curve is indicative of a thermally stable sample. DTG curve provided helpful data for examining the behavior of NCQDs and also determining the induced changes in the sample by evaluating its peaks.

*Dynamic light scattering (DLS) and zeta potential*

We exerted the technique of dynamic light scattering (DLS) to estimate the particle size of NCQDs by the usage of a Zeta sizer-Nano ZS90 (Malvern Instruments, UK) at ambient temperature.

The surface charge of the sample was measured by determining the Zeta potential, also known as ζ potential, which was achieved through the application of Zeta sizer-Nano ZS90 (Malvern Instruments, UK) at 25 ^°^C. It should be further noted that Zeta potential can directly affect the nano-particles stability.

*Electron microscopics*

The NCQDs images were acquired from Field Emission Scanning Electron Microscopy (FESEM, TESCAN MIRA 3, Czech Republic) for analyzing the obtained morphological structure. In addition, the element of prepared NCQDs was characterized by Energy Dispersive X-rays spectroscopy (EDX), which contained an accelerating voltage of 15 kV for 30 min to identify the elemental distribution (such as S, O, N and C) of the sample. In this technique, the Au element was exerted for coating in order to prepare the images.

The morphology (disperse formation and shape) of prepared NCQDs was investigated through the operation of transmission electron microscopy (TEM, Zeiss-EM10C, Germany) at 100 kV. For this purpose, the sample suspension was diluted with distilled water and sonicated with a Misonix sonicator (Misonix- S3000, USA) for 10 min. Then, a specified part of the sample (20 μl) was transferred in the form of carbon film on a copper grid 300 mesh (EMS-USA) to be dried for 1 hr at ambient temperature.

A drop of diluted NCQDs suspension (0.2 wt.%) was cast on a glass cover and vacuum dried. The images were obtained using AFM (Park system, USA) in the tapping mode with a scanning frequency of 150 kHz. The AFM had silicon nitride (Si**₃** N**₄**) tips with a curvature radius of 10 nm with a cantilever of force constant (42 N/m) and spring constant (1 N/m). The scanning areas were 2.0×2.0 μm with an image resolution of 512×512 pixels. AFM observations were carried out in duplicate for each material, and the average diameter of NCQDs was acquired from the height profiles of over 52-grain particles using Pico Nanoscope software (Park system, USA). The morphology, dimension, and roughness of the synthesized NCQDs were investigated through the utilization of AFM**.** The 2D and 3D topographical images were measured by the tapping functional mode that involved the usage of a needle tip with a radius of 10 nm.

*Optical properties*

In this section, we studied the obtained UV-Vis absorption and fluorescence PL spectra for identifying the optical properties of the solution that contained natural carbon quantum dots.

In order to measure the UV-visible spectroscopy, the utilization of a V-630 UV-visible spectrophotometer (Jasco, Japan) through the absorption wavelength of 200-800 nm was considered.

The photoluminescence (PL) emission intensity, as the function of excitation, was recorded by (F-2500, Hitachi, Tokyo, Japan).

We measured the quantum yield of NCQDs by using the solution of quinine sulfate as a standard after being dissolved in 0.1 M of H_2_SO_4 _with a fluorescence quantum yield of 0.54 at 360 nm, η_=_1.33. The QY of NCQDs was calculated through the following equation (Eq. 2): 


 (2) $$Q_{\mathrm{CQDs}}=Q_{\mathrm{st}}\frac{(I_{\mathrm{CQDs}})(A_{\mathrm{st}}){\left(n_{\mathrm{CQDS}}\right)}^{2}}{(I_{\mathrm{st}})(A_{\mathrm{CQDs}}){\left(n_{\mathrm{st}}\right)}^{2}}$$

Where Q is the fluorescence quantum yield, I represents the emission intensity, η would be the refractive index of solvent, and A stands for the absorbance in the sample; the subscript “st” and “CQDs” represent the standard and carbon quantum dots, respectively.

*MTT assay*

The importance of studying the cytotoxicity of synthesized NCQDs is quite undeniable due to their potential applications in medical therapy, bio-imaging, etc. The cellular compatibility tests were conducted on Mouse Colon Carcinoma (C26) cells, which were cultured in Dulbecco’s Modified Medium (DMEM) for performing an incubation process at 37 ^°^C and 5% CO_2_. Once the medium was shifted, the product was confronted with increasing concentrations of NCQDs. The next step included another incubation process at 37 ^°^C in 5% CO_2_ for a period of 24 hr. It should be mentioned that this study included a blank and a positive control sample as well. The employment of a Synergy™ HT Multi-Mode Microplate Reader (Biotek Instruments, Winooski, VT, USA) at 570 nm was considered to analyze the toxicological characterization.

***Detection of Ca*** ^2+^ ***and Zn*** ^2+^

The following is the procedure to detect Ca^2+^ and Zn^2+^ ions using NCQDs. First, NCQDs solution in a concentration of 2 µg/ml was prepared by dissolving 0.5 mg NCQDs in de-ionized water in a 250 ml volumetric flask. Then 2.0 ml NCQDs solution so prepared was transferred into a quartz-cuvette and certain volumes of Ca^2+^ and Zn^2+^ solutions were added. Subsequently, the emission spectra were measured in an excitation slit of 5 nm and an emission slit of 10 nm.

***Trypsin activity determination***

Trypsin activity was assessed without and with the application of NCQDs by using a UV-Vis spectrophotometer (Jasco V-630, Japan) with 1 cm quartz cells. The measurement of enzyme activity required the exertion of BApNA as the substrate, which can be degraded into *p*-nitro-aniline by the presence of trypsin in the solution (49-51). The applied reaction is provided in the following:

BApNA (colorless) + trypsin <=> *p*-nitroaniline (yellow) + arginine + benzoic acid + trypsin.

In order to evaluate the activity of trypsin as a control (in the absence of NCQDs), different concentrations of BApNA, trypsin (0.1 mg/ml), and phosphate buffer were completely mixed and incubated (37 ^°^C for 10 min). The same experiments were performed with two concentrations of NCQDs (50 µg.ml^-1^ and 100 µg.ml^-1^) for exploring their possible effects on trypsin activity. The absorbance of the samples was determined at 410 nm to evaluate the trypsin activity in different conditions. Plots were drawn by the exertion of GraphPad Prism 8 software.

## Results

The prepared NCQDs with a high quantum yield (26.8%) were synthesized through a facile hydrothermal method that involved the usage of orange pericarp. 

***Chemical analysis***

**Figure 1:**
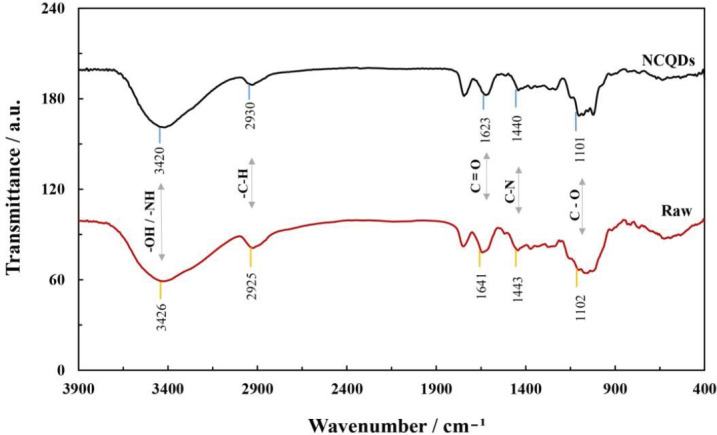
FTIR spectra of the raw and natural carbon quantum dots (NCQDs) samples

**Figure 2:**
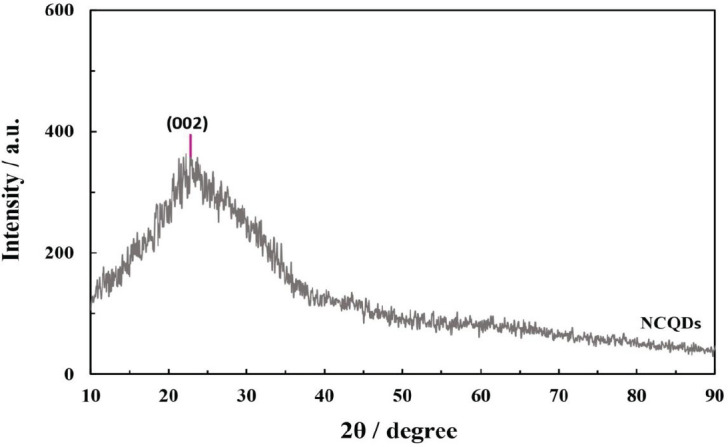
XRD pattern of the synthesized natural carbon quantum dots (NCQDs)

As displayed in Figure 1, the functional groups that exist on the surface of prepared NCQDs were investigated by FTIR spectra analysis. The observed peaks at 1440 and 1443 cm^-1^ are associated with C-O and the peaks detected at 1623 and 1641 cm^-1^ are apparently corresponding to C=O. Meanwhile, the peaks that can be perceived at 2930 and 2925 cm^-1^ can be assigned to the stretching vibration of C-H. The O-H and N-H groups were identified from the peaks at around 3420 and 3426 cm^-1^. According to Figure 2, a broad diffraction peak at around 22.78^°^ was observed, which was consistent with the crystal lattice (002) plane of carbon-based materials and corresponded to the amorphous carbon of orange pericarp (52). 

***Thermal analysis***

**Figure 3:**
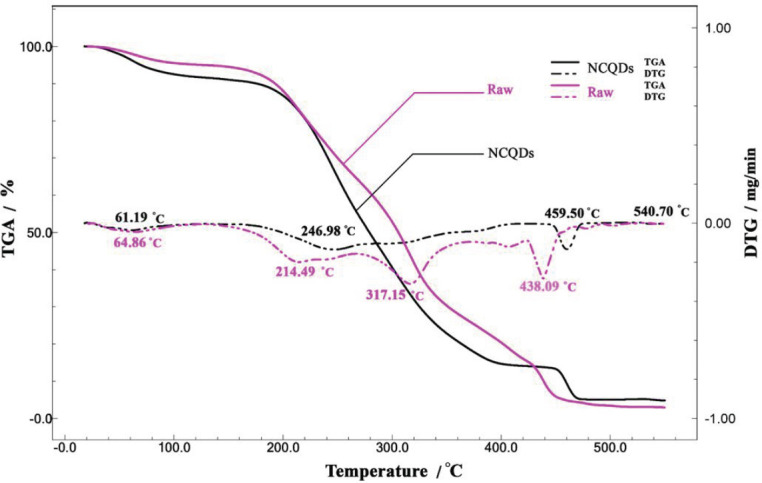
TGA and DTG curves of the raw and NCQDs samples under an air atmosphere at 10 ^°^C/min heating rate

TGA: Thermogravimetric analysis; DTG: Derivative thermogravimetric; NCQDs: Natural carbon quantum dots

The thermal gravimetric analysis (TGA/DTG) of prepared NCQDs and raw sample, along with the observed properties, is represented in Figure 3. The TGA curve displayed three significant weight losses (changes in sample composition, thermal stability, and kinetic parameters) that usually occur due to chemical reactions and physical transitions. The capillary pore residual water evaporated between the temperature range of 61 ^°^C and 100 ^°^C. In addition, the rate of mass alteration was indicated by the DTG curve. The samples were heated throughout the region of ~ 20–550 ^°^C, at a heating rate of 10 °C/min. The decomposition process of NCQDs was observed to begin at around 400 ^°^C and reach up to 500 ^°^C. The decomposition peak gained the maximum rate of mass loss at 438.09 ^°^C and 459.50 ^°^C for the cases of raw sample and the prepared NCQDs, respectively, and it can be indicated, there is higher stability in the case of NCQDs.

***DLS and zeta potential***

**Figure 4:**
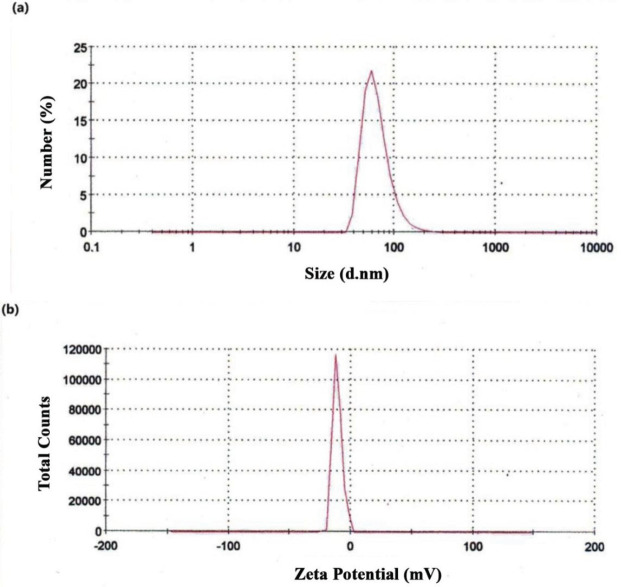
(a) Dynamic light scattering (DLS) and (b) Zeta potential value of NCQDs

Figure 4a displays the estimated Z-Average of the prepared NCQDs that was obtained through the technique of dynamic light scattering (DLS). According to the DLS data, the NCQDs contained a size of about 67 nm. In addition, the electrical charges of nano-particles surfaces and their colloidal stability were distinguished by performing a zeta potential analysis. The surface charge of synthesized NCQDs is exhibited in Figure 4b. The value of NCQDs zeta potential was observed to be -11 mV, while this negative value can confirm the satisfying colloidal stability of this product (53).

***Particle size and morphology study***

**Figure 5:**
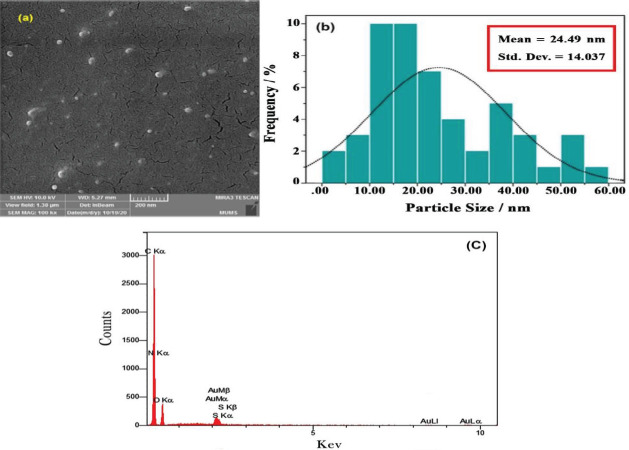
FESEM image (a), particle distribution (b), and EDX analysis (c) of NCQDs

FESEM: Field Emission Scanning Electron Microscopy; EDX: Energy dispersive x-ray spectroscopy; NCQDs: Natural carbon quantum dots

The obtained highly magnified FESEM image of NCQDs is displayed in Figure 5a. As it can be observed, nano-particles were thoroughly dispersed throughout the sample and according to Figure 5b, the range of particle sizes is 24±14 nm while the spherical appearance of synthesized NCQDs can be detected. It is indicated by the EDX results (Figure 5C) that the C, N, O, and S elements are dispersed evenly on the surface of NCQDs. The A% of C, N, O, and S were found to be 72.15, 3.18, 18.24, and 0.44 %, respectively.

**Figure 6:**
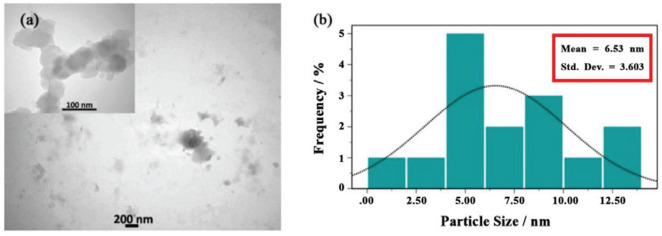
(a) TEM images of natural carbon quantum dots (NCQDs) synthesized from orange pericarp. (b) Particle size distribution curve of the NCQDs

**Figure 7:**
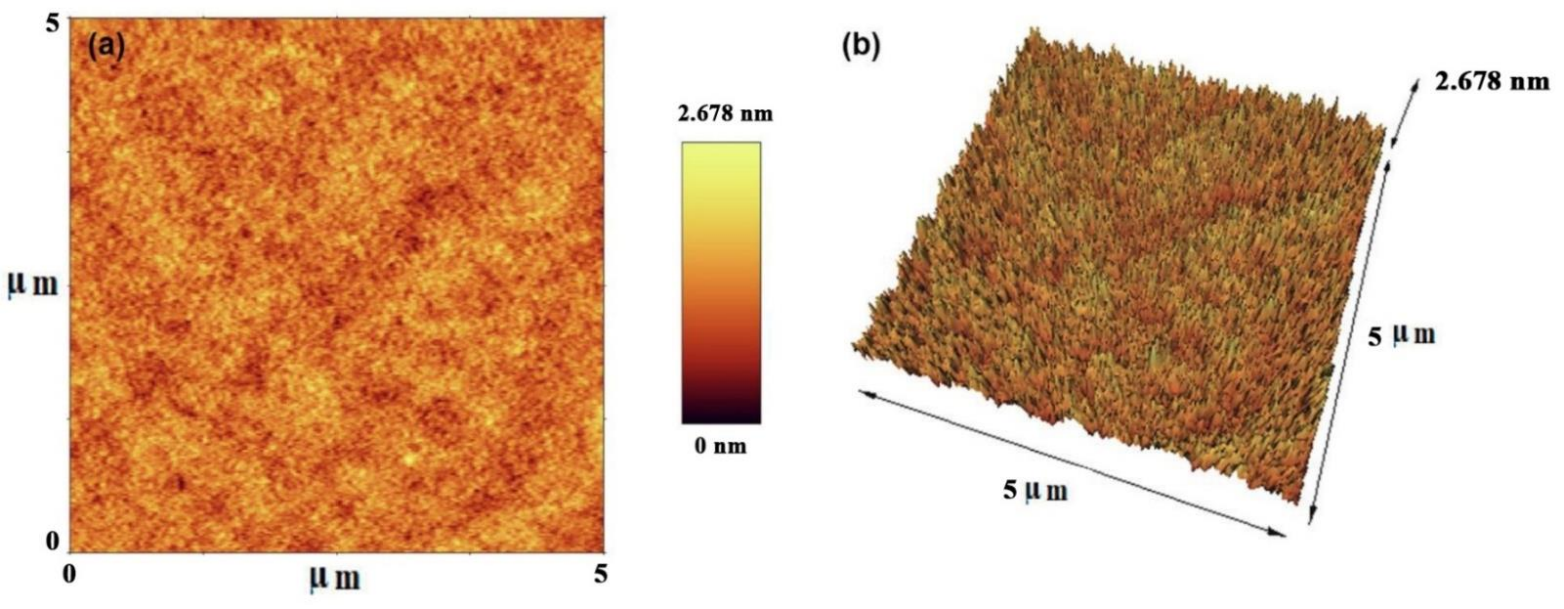
AFM images of prepared natural carbon quantum dots (NCQDs) (scan area 5 µm × 5 µm); a-2D, b-3D

The provided TEM images in Figure 6a present an examination of the shape and size of NCQDs in different scales (200 and 100 nm). Accordingly, the prepared NCQDs contained a spherical shape with satisfying dispersity. In addition, the particle size distribution of NCQDs is displayed in Figure 6b. The average size was determined to be around 6–7 nm. Furthermore, AFM images provided the morphology and topography of our samples. The two-dimensional (a) and three-dimensional (b) images of prepared NCQDs are exhibited in Figure 7. Beside the provided image in Figure 7a, the color gradient was indicative of an induced topographic difference (2.678 nm), in which a low difference can confirm the good dispersion of NCQDs. Also, the spherical shape, dimension, and surface roughness of our sample can be observed in Figure 7b.

***Optical properties***

**Figure 8:**
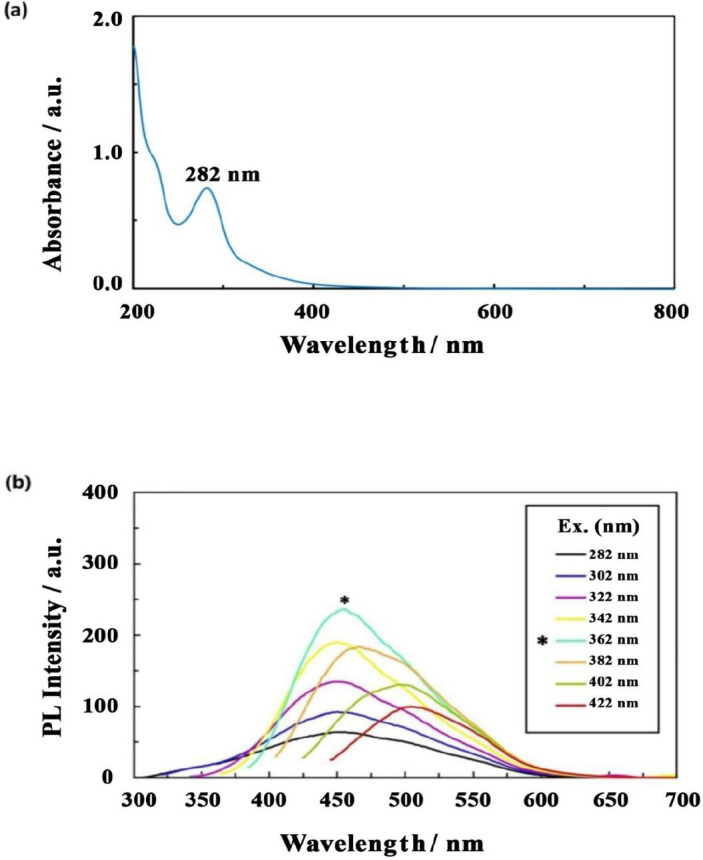
(a) UV-visible absorption spectra of NCQDs. (b) PL emission spectra of NCQDs at various excitation wavelengths from 282 nm to 422 nm

NCQDs: Natural carbon quantum dots; PL: Photoluminescence

Figure 8 displays the UV-visible absorption and PL spectra technique that was exerted to evaluate the optical features of synthesized NCQDs. According to Figure 8a, the absorption peak at 282 nm can be related to the π that is in correspondence with the carbon core C=C units transition *π- (54). Figure 8b exhibits the PL spectra of NCQDs, which were attained at varying excitation wavelengths of 282-422 nm. The PL emission of CQDs commonly relies on the excitation wavelength and is regularly decreased as this particular factor is increased. The PL emission spectra wavelength of NCQDs was observed to reach the peak at 454 nm with an excitation wavelength of 362 nm. 

***Cytotoxicity***

**Figure 9:**
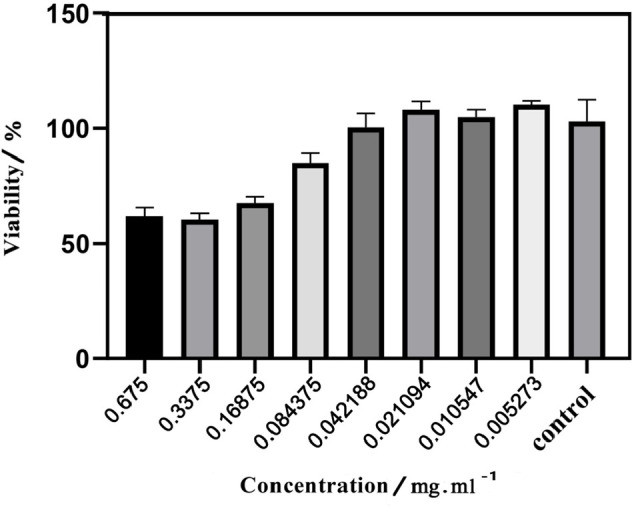
Results from cytotoxicity evaluation of the synthesized NCQDs on C26 cells estimated by MTT assay after exposure to different concentrations for 24 hr

NCQDs: Natural carbon quantum dots; MTT: 3-(4,5-dimethylthiazol-2-yl)-2,5-diphenyl-2H-tetrazolium bromide

The MTT assay was performed on mouse colon carcinoma (C26). The cytotoxicity results of synthesized NCQDs are demonstrated in Figure 9. After 24 hr of treatment, the cell viabilities were examined based on being exposed to different concentrations of NCQDs (0.005273, 0.010547, 0.021094, 0.042188, 0.084375, 0.1687, 0.3375, and 0.675 mg/ml) (55). According to the results, the green-CQDs exhibited fine biocompatibility in the concentration range of 0.005273 to 0.084375 mg/ml (viability over 90 %). It was observed that the viability percentage of NCQDs faces a decrease as the concentration is increased. 

***Detection of Zn*** ^2+^ ***and Ca*** ^2+^

**Figure 10:**
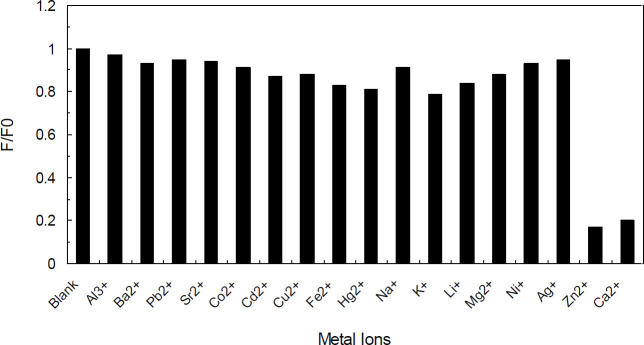
Effect of different cations (at 0-1.0 mM) on the emission intensity of the natural carbon quantum dots (NCQDs)

**Figure 11:**
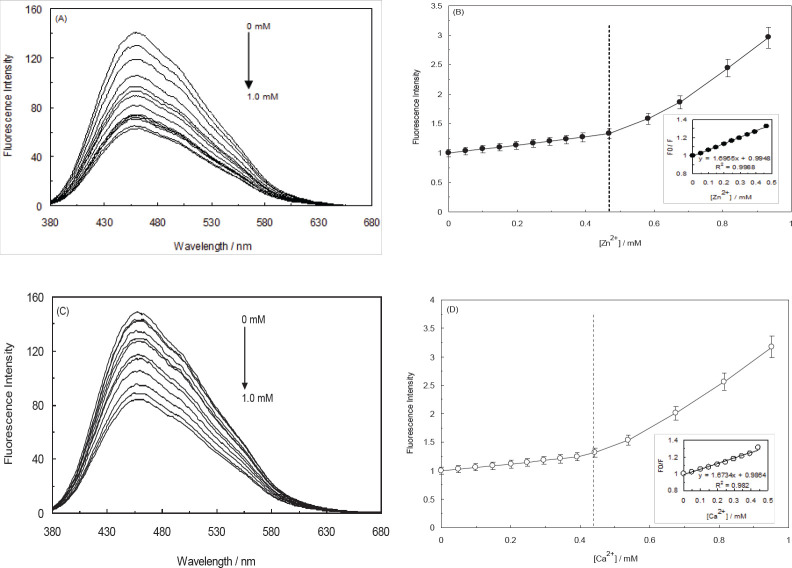
(A, C) Effect of different concentrations of Zn^2+^ and Ca^2+^ on the fluorescence intensity of the natural carbon quantum dots (NCQDs) by 365 nm excitation wavelength. (B, D) relationship between F0/F and the concentration of Zn^2+^ and Ca^2+^. Insets show a linear relationship within the range of 0-0.5 mM

Figure 10 displays the results of monitoring the fluorescent spectra of N-CQDs under a slight disturbance of cations. The PL intensity of N-CQDs solutions (2 µg/ml), which implicated the addition of different cations (Al^3+^, Ba^2+^, Pb^2+^, Sr^2+^, Co^2+^, Cd^2+^, Cu^2+^, Fe^2+^, Hg^2+^, Na^+^, K^+^, Li^+^, Mg^2+^, Ni^+^, Ag^+^, Zn^2+^, and Ca^2+^) in a concentration of 1.0 mM, was observed to be quenched more than 95% by Zn^2+^ and Ca^2+^; considering this data, this product can be suggested as a superior fluorescent probe for the detection of Zn^2+^ and Ca^2+^ fluorescence. In conformity to Figure 11A and 11C, the PL intensity was monotonously decreased as the concentrations of Zn^2+^ and Ca^2+^ were enhanced. 

A possible mechanism of N-CQDs fluorescent quenching by Zn^2+^ and Ca^2+^ was investigated through the application of the Stern-Volmer equation (56):

F_0_/F = 1 + Ksv[Q] (3)

Where Ksv is the quenching constant, [Q] refers to the concentrations of the quenching agent, and F and F_0_ stand for the fluorescent intensities of NCQDs with and without the quenching agent, respectively. As demonstrated in Figures 11B and 11D, the factor of F_0_/F against the concentration of Zn^2+^ and Ca^2+^ can be linearly fitted in the concentration limitation of 0–0.5 mM. The obtained Ksv values of NCQDs upon the binding of Zn^2+^ and Ca^2+^, which were 1.69×10^3^ M^-1^ and 1.67×10^3^ M^-1^, respectively, were indicative of an obvious static quenching mechanism (56). On the other hand, the Ksv values revealed higher sensitivity of the applied NCQDs for Zn^2+ ^detection than that of the Ca^2+^.

***Assay of trypsin activity in the presence of NCQDs***

**Figure 12:**
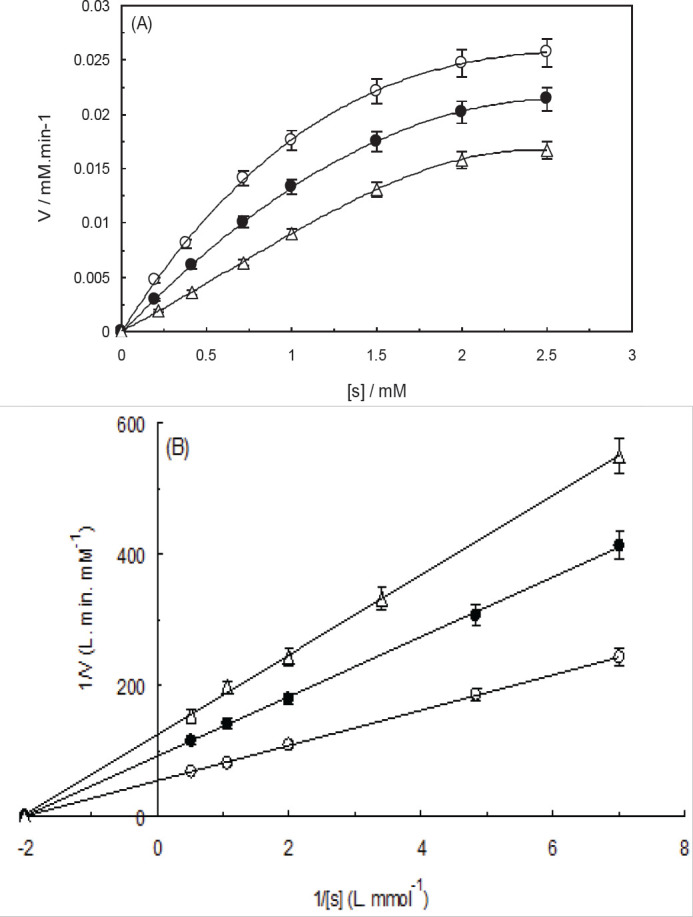
(A) Michaelis-Menten curve derived from trypsin activity data at various natural carbon quantum dots (NCQDs) concentrations. (B) Double-reciprocal plot of the Michaelis-Menten curve. Conditions: C_NCQDs_: 0 μg.ml^-1^ (Open circles), 50 μg.ml^-1^ (closed circles) and 100 μg.ml^-1^ (Open triangle), pH: 7.5, T: 298 K, C_trypsin_: 0.1 mg/ml

We performed enzyme activity analyses to examine the influence of NCQDs on the catalytic factors of trypsin in the presence of BApNA as a substrate (0-2 mM). Figure 12A exhibits the Michaelis-Menten graph of enzyme reactions with and without applying the two different concentrations of NCQDs. According to this figure, NCQDs (50 µg.ml^-1^ and 100 µg.ml^-1^) can reduce the action velocity of enzymes under the same substrate concentrations (V). The decreased activity of trypsin as the main digestive protease may result in reducing the rate of nutrient intake. The inhibition model of NCQDs was evaluated through the usage of the Lineweaver-Burk diagram (Figure 12B). According to the data presented in Table 1 and Figure 12A, a considerable decline was observed in the V_max_ (trypsin’s maximum rate) values after the addition of two NCQDs concentrations, while the K_m_ (trypsin’s affinity for BApNA) values remained nearly constant. According to the results, NCQDs inhibition was non-competitive for trypsin. In this inhibition mechanism, the V_max_ values of the enzyme progressively decreased while the K_m_ values remained stable. The enzymatic capabilities of trypsin were indicated by its catalytic efficiency (k_cat_/K_m_). k_cat _is the catalytic constant that can be calculated through the k_cat_ = V_max_/[E_t_] formula (57). According to Table 1, the values of k_cat_ and k_cat_/K_m_ faced a decrease as a result of increasing the concentration of NCQDs. It is suggested by these findings that NCQDs can alter the spatial structure of trypsin (based on experimental spectroscopic data). These structural changes caused a reduction in the rate of trypsin activity.

## Discussion

Natural carbon quantum dots (NCQDs) are known as members of the novel nano-particles family that contains sizes smaller than 10 nm. In this study, the synthesis of natural carbon quantum dots by the usage of orange pericarp as the carbon natural resource for the very first time has been performed.

According to the chemical analysis, the presence of hydroxyl, carboxyl, and amino groups at the surface of CQDs was proved by the provided data from FTIR spectra (58, 59). The appearance of these groups is indicative of the excellent water-solubility and biocompatibility of CQDs (60). Furthermore, the thermal gravimetric analysis of prepared NCQDs and raw samples has been done. We were able to calculate the weight % of every induced mass alteration as the samples’ varying components were splintered as a result of heightening the temperature. The mass unity of any sample of this type would be disturbed in the course of being processed at high temperatures (61).

In order to study the hydrodynamic diameter in colloidal solution, dynamic light scattering (DLS) measurements have been applied. The size of NCQDs has been obtained at about 67 nm, which may be due to the agglomeration of the particles. The stability of the synthesized NCQDs and the surface charge have been shown throughout the Zeta potential analysis. The obtained negative value of zeta potential represents the negatively charged carboxyl and hydroxyl functional groups on the surface of NCQDs, and the colloidal stability of the product has been confirmed.

Field-scanning electron microscopy (FESEM) analysis was conducted to distinguish the morphology and construction of CQDs (62). The dispersion of nano-particles throughout the sample has been demonstrated by FESEM measurements. As can be shown through the energy dispersive electron (EDX) analysis, the peaks of carbon is the main element composition in NCQDs, and the peak of nitrogen, oxygen, and sulfur are probably originated from the substrate. As a result, the successful synthesis of CQDs was confirmed by observing the fact that the highest A% belongs to the element (63). Furthermore, we were able to observe the morphology and topography of our samples through the attained AFM images.

The gathered data on PL displayed the appearance of red-shifted wavelengths with reduced emission intensity, which was in correlation to the previous reports, in which CQDs manifested an emission pattern that relied on excitation (64-66). The PL intensity of N-CQDs is extremely sensitive to certain minute perturbations and therefore, they can be an excellent candidate for the detection of certain perturbations. 

MTT assay study showed very low toxicity of CQDs was detected between the viability percentage ranges of 70%–60%, which was related to the fact that nano-particles can take up the cells in high concentrations (0.16875–0.675 mg/ml). The reported low cytotoxicity can be considered proof of the applicability of fluorescent CQDs for being applied in bio-imaging implementations and cancer therapy (67, 68). The obtained data from Ksv values and the static quen

ching mechanism have been confirmed.

Our findings on enzyme activity revealed the disturbed situation of trypsin’s structure, while its activity is likely to be affected by these changes. Based on different studies, the catalytic activity of enzymes can be influenced by various factors such as the loss of critical residues from the enzyme surface, alterations in the enzyme’s structural integrity, and the change in the enzyme’s microenvironment (69, 70). The decreased activity of trypsin as the main digestive protease may result in reducing the rate of nutrient intake.

**Table 1:** Kinetic parameters of trypsin in the absence and presence of natural carbon quantum dots (NCQDs), at 310 K and pH 7.5

[NCQDs] ( g.ml^-1^)	V_max_ (mM.min^-1^)	K_m_ (mM^-1^)	k_cat_ (min^-1^)	kcat/K_m_ (mM^-1^.min^-1^)
0	0.0236	0.7040	54.83	77.88
50	0.0040	0.6698	10.00	14.29
100	0.0021	0.6681	5.01	7.50

## Conclusion

In this experiment, we ensured the biocompatibility of synthesized NCQDs, which belong to the nano-materials family. Nowadays, the application of green, low-cost, and economical methods for environmental protection stands as a necessity. According to the provided outcomes, we were able to successfully synthesize and achieve stable NCQDs for the very first time, by the usage of orange pericarp as a carbon precursor through a simple hydrothermal method. The prepared NCQDs were characterized through FTIR, XRD, TGA, DLS, Zeta Potential, EDX, FESEM, TEM, AFM, UV-visible, and PL spectra analyses. This product was observed to contain high photo-stability, excellent PL activity, and efficient fluorescent emission based on excitation. The strong peak of fluorescence absorption and excitation wavelength exhibited a high quantum yield of 26.8%. According to the performed TEM analysis, the average size of these NCQDs was found to be in the range of 6-7 nm along with a spherical shape. The satisfying colloidal stability of this product was ensured by zeta potential results while the existence of functional groups on the surface of prepared NCQDs was confirmed by FTIR spectroscopy. In addition, there was no cytotoxicity observed throughout the MTT assay on C26 cells with regard to cellular toxicity effects. Zn^2+^ and Ca^2+^ ions have been efficient quenchers of the NCQDs emission; therefore, NCQDs are good candidates for fluorescent probes of Zn^2+^ and Ca^2+^ ions. The V_max_ values of trypsin in the presence of NCQDs changed, while the K_m_ values remained nearly constant which showed NCQDs inhibition was non-competitive for trypsin. In summary, we introduced a novel, strong, and safe procedure for the production of green NCQDs that can be applied for multipurpose applications. The hydrothermal methods for preparing CQDs have thermal limitations that do not respond for all natural compounds and should be considered by scientists, on the other hand, enzyme activity assay for some enzymes is difficult and expensive and should be considered to find new methods for enzyme activity assay.

## Authors’ Contributions

STSY, NS, SH, and PM Performed the research and collected the data. S TSY, BMN , and JC Analyzed the data. STSY wrote the initial draft of the manuscript. JC Revised the manuscript. All authors discussed the results and contributed to the final manuscript.

## Conflicts of Interest

The authors declare their lack of competing financial interests or personal relationships that could have appeared to influence the work reported in this paper.
